# Immunotherapy-associated autoimmune hemolytic anemia induced by anti-PD-1 therapy in esophageal cancer: A case report and literature review

**DOI:** 10.1097/MD.0000000000042174

**Published:** 2025-04-11

**Authors:** Zijian Qiu, Mixue Sun, Chunyan Dai, Xiaoping Zhu

**Affiliations:** a Department of Radiation Oncology, The Quzhou Affiliated Hospital of Wenzhou Medical University, Quzhou People’s Hospital, Quzhou, Zhejiang, China; b Inpatient Ward of 351, The Quzhou Affiliated Hospital of Wenzhou Medical University, Quzhou People’s Hospital, Quzhou, Zhejiang, China.

**Keywords:** autoimmune hemolytic anemia, case report, esophageal cancer, immunotherapy, sintilimab

## Abstract

**Rationale:**

Numerous immune checkpoint inhibitors have been approved for clinical use in metastatic advanced esophageal cancer. While immunotherapy brings therapeutic benefits, immune-related adverse events (irAEs) should nevertheless not be overlooked. This paper reports on the first documented case of Autoimmune hemolytic anemia (AIHA) caused by anti-programmed cell death protein-1 therapy in esophageal squamous cancer.

**Patient concerns:**

An 84-year-old female patient with metastatic squamous esophageal cancer developed chest tightness, generalized weakness, and a yellowing of the skin after 2 cycles of sintilimab treatment.

**Diagnoses:**

Initial examination revealed severe anemia with elevated levels of bilirubin, reticulocytes, lactate dehydrogenase, decreased levels of haptoglobin, and a positive direct antihuman globulin test. The patient was diagnosed with immunotherapy-associated AIHA.

**Interventions:**

The patient was promptly treated with corticosteroids and human immunoglobulin, supportive transfusion with washed erythrocytes.

**Outcomes:**

Her AIHA was controlled after treatment. Subsequent immunotherapy was not continued, and there was no recurrence of AIHA.

**Lessons:**

We have identified a rare case of serious adverse reaction caused by anti-PD-1 therapy. We summarize the clinical presentations, diagnosis, and treatment of this case of immunotherapy-related AIHA and discuss the pathogenesis and therapeutic advances in immunotherapy-related AIHA, as well as sintilimab-induced irAEs, in detail. These findings underscore the importance of the early detection, diagnosis, and treatment of these rare and potentially fatal irAEs.

## 1. Introduction

China has one of the highest global incidence and mortality rates for esophageal cancer, with approximately 85% of cases being squamous cell carcinomas.^[1]^ Unresectable recurrent or metastatic esophageal cancer shows a limited response to chemotherapy and is associated with poor outcomes. The advent of immune checkpoint inhibitor (ICI) therapies has improved the survival rates of patients with recurrent or metastatic esophageal cancer.^[2]^ The current National Comprehensive Cancer Network guidelines recommend anti-programmed cell death protein-1 (PD-1) therapy (pembrolizumab^[3]^ or nivolumab^[4]^) combined with chemotherapy as the preferred regimen for recurrent or metastatic advanced esophageal cancer. The ORIENT-15 study^[5]^ conducted in China showed that first-line sintilimab combined with chemotherapy enhanced the progression-free survival and overall survival of patients with advanced or metastatic esophageal squamous carcinoma (ESC). As a result, this regimen received a level I recommendation based on class 1A evidence in the Chinese Society of Clinical Oncology guidelines.

Immunotherapy-related adverse events (irAEs) can occur in any organ system, and common adverse reactions include dermatitis, endocrine hypofunction, diarrhea, and pneumonia.^[6]^ Although hematologic irAEs are rare (about 3%–4% of total irAEs), severe hemopenia can be fatal.^[7]^ Sintilimab is a PD-1 inhibitor developed in China. While its indications continue to expand, attention must also be given to its adverse drug reactions, particularly rare and severe irAEs. Thus far, there have been no reports on sintilimab-induced autoimmune hemolytic anemia (AIHA). Here, we describe the diagnosis and treatment process of a patient with metastatic ESC who developed AIHA after receiving sintilimab in detail.

## 2. Case presentation

An 84-year-old Chinese female patient with ESC was hospitalized on January 19, 2024 due to chest tightness, weakness, and a yellowing of the skin for 1 week. She denied having any other symptoms, including fever, cough, abdominal pain, or bleeding. She had a past history of hypertension, atrial fibrillation, and coronary artery disease (after percutaneous coronary intervention), as well as a medication history of amlodipine besylate, rivaroxaban, metoprolol tartrate, atorvastatin calcium, furosemide, and spironolactone tablets, with no known drug allergies.

Upon reviewing her medical history, the patient was found to have been diagnosed with mid-ESC (cT3N0M0, stage II) 8 years prior (January 2016), having received radical radiotherapy (60 Gy/30 F) at that time without concurrent chemotherapy or targeted therapy. Then, the patient received 2 courses of oral chemotherapy with tegafur, gimeracil, and oteracil potassium capsules (TGOPC) after the completion of radiotherapy. She was followed up at our outpatient clinic and her tumor was assessed as stable. In October 2023, the patient’s esophageal obstruction worsened, and gastroscopy revealed esophageal neoplasm. Positron emission tomography/computed tomography demonstrated a thickening of the lower and middle esophageal wall with increased fluorodeoxyglucose uptake, indicative of ESC recurrence. Multiple enlarged lymph nodes in the mediastinum, abdomen, and pelvis with increased fluorodeoxyglucose uptake were also noted, suggesting metastases. The patient was diagnosed with ESC recurrence with systemic metastases (stage IV) at the time. The patient was immediately treated with 2 cycles (December 1, 2023 and December 25, 2023) of TGOPC (40 mg, per os, twice a day, day 1–14) combined with sintilimab (200 mg, intravenous drip, day 1).

**Figure 1.**
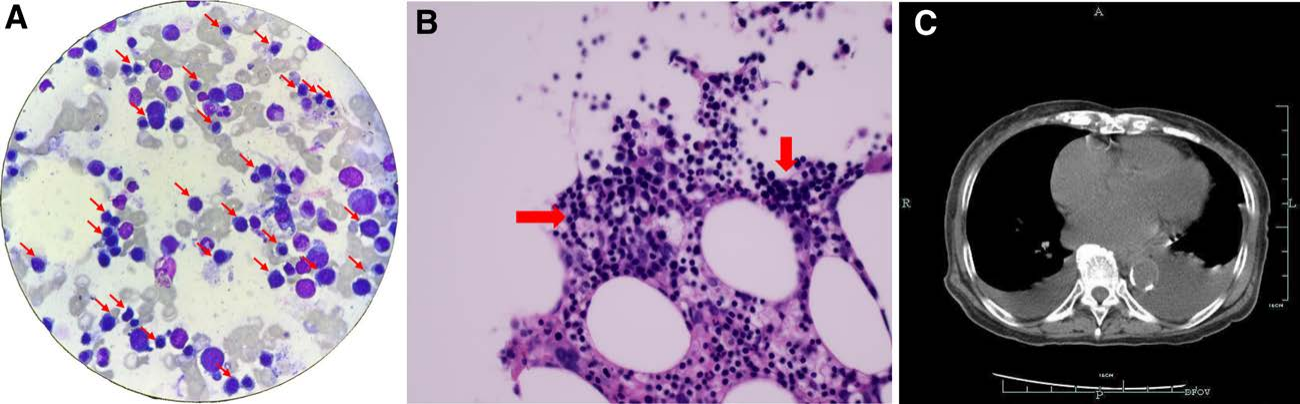
Bone marrow and imaging findings of the patient. (A) Bone marrow smear findings of marked erythroid hyperplasia (the erythroblasts marked by the red arrows). (B) Bone marrow biopsy findings of moderately active bone marrow erythroid hyperplasia (the area marked by the red arrows). (C) Imaging findings indicative of a moderate amount of bilateral pleural effusion.

Laboratory tests on the day of admission showed hemoglobin 55 g/L, red blood cell count 1.41 × 10^12^/L, reticulocytes 15.24%, lactate dehydrogenase 308.2 U/L, total bilirubin 335.7 μmol/L, indirect bilirubin 294.2 μmol/L, platelet count 170 × 10^9^/L, white blood cell count 7.2 × 10^9^/L, creatinine 105.2 μmol/L, B-type natriuretic peptide 691.30 pg/mL, and positive direct antihuman globulin test (Coombs test) [anti-IgG, C3d]. Further investigations revealed free hemoglobin 70.15 mg/L, haptoglobin < 25 mg/dL, bone marrow smear findings of marked erythroid hyperplasia, bone marrow biopsy findings of moderately active bone marrow erythroid hyperplasia, and imaging findings indicative of a moderate amount of new bilateral pleural effusion. Details of these findings are summarized in Table 1 and Figure 1. As a result, we diagnosed the patient with AIHA and immediately discontinued subsequent sintilimab treatment.

**Table 1:** Laboratory findings at the time of diagnosis of AIHA and the time of significant improvement in the condition.

Investigation	Pretreatment result on January 19, 2024 [normal range]	posttreatment Result on February 1, 2024 [normal range]
Hemoglobin (g/L)	55 [115–150]	90 [115–150]
White cell count (×10^9^/L)	7.2 [4.0–10.0]	7.4 [4.0–10.0]
Platelets (×10^9^/L)	170 [100–300]	51 [100–300]
Lactate dehydrogenase (U/L)	308.2 [109.0–245.0]	222.5 [109.0–245.0]
Total bilirubin (μmol/L)	335.7 [5.1–20.5]	21.9 [5.1–20.5]
Indirect bilirubin (μmol/L)	294.2 [0.0–6.8]	9.5 [0.0–6.8]
Reticulocyte count (%)	15.24 [0.40–1.60]	3.89 [0.40–1.60]
Haptoglobin (mg/dL)	<25 [30.00–200.00]	103.58 [30.00–200.00]
Plasma free hemoglobin (mg/L)	70.15 [<40.00]	<25.00 [<40.00]
Coombs test	Positive (IgG)	Negative (IgG)

AIHA = autoimmune hemolytic anemia.

**Figure 2.**
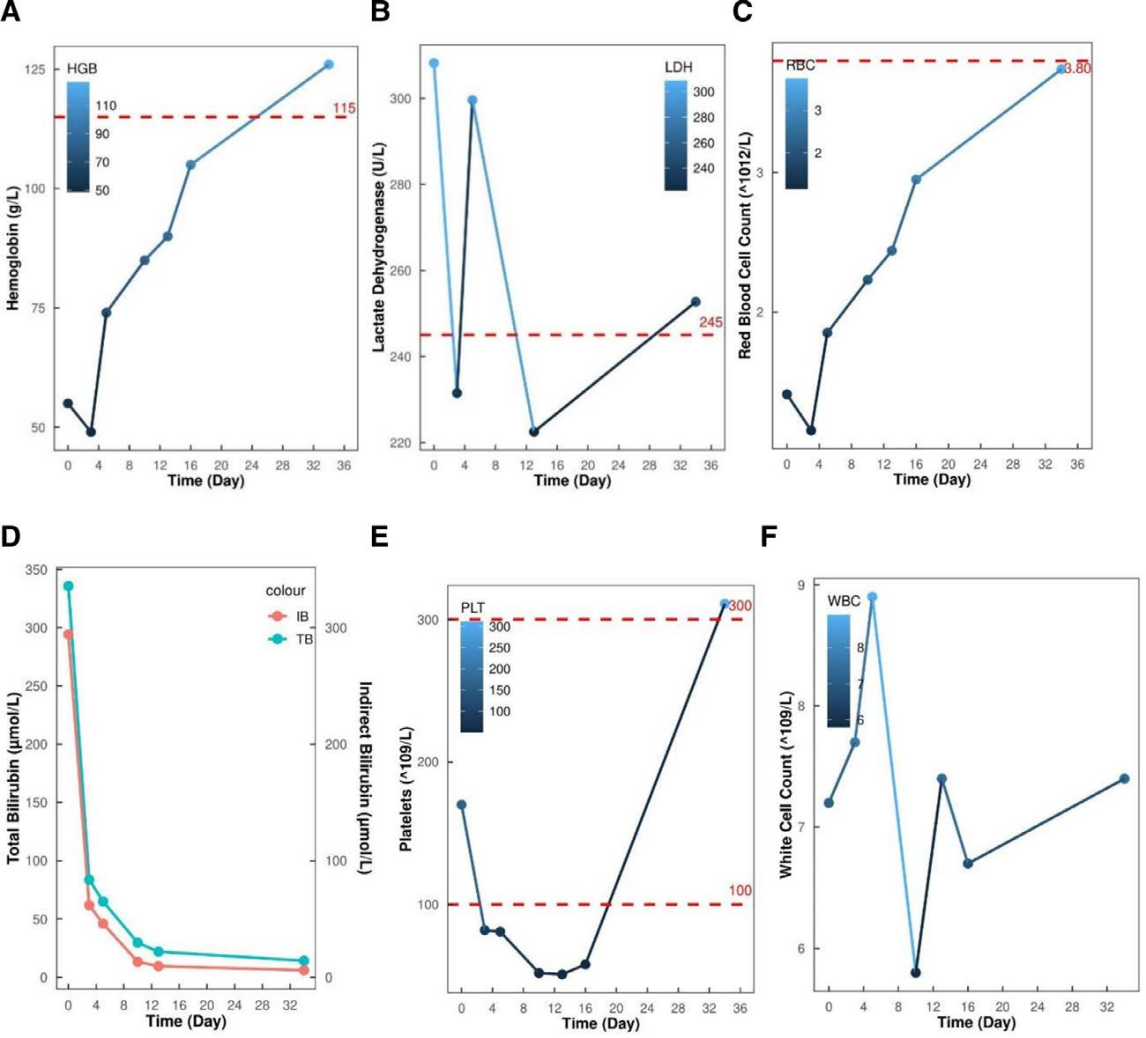
Dynamic hematology tests during the treatment period. (A) Hemoglobin (g/L). (B) Lactate dehydrogenase (U/L). (C) Red blood cell count (×10^12^/L). (D) Bilirubin (μmol/L). (E) Platelets (×10^9^/L). (F) White cell count (×10^9^/L).

Promptly after the diagnosis was confirmed, the patient was administered 80 mg of methylprednisolone (intravenous injection, once a day) for 4 days, followed by 60 mg (once a day) for 4 days and 40 mg (once a day) and 30 mg (once a day) for 5 days each. Additionally, the patient was concomitantly administered immunotherapy with 17.5 g of human immunoglobulin (PH4; 0.4 g/kg/d, intravenous drip, once a day) for 5 days, supportive transfusion with 2 U of washed erythrocytes, and thoracentesis for pleural effusion drainage. The patient’s symptoms of chest tightness were evident. Dynamic hematology tests during the treatment period (Fig. 2) showed a progressively elevated red blood cell count and progressively decreased serum bilirubin levels. Furthermore, an improvement in the patient’s mental status was noted. The laboratory test results during the treatment period are summarized in Table 1. The patient developed thrombocytopenia during hospitalization and was administered 15,000 U of subcutaneous recombinant human thrombopoietin injection and oral herombopag as a platelet-boosting therapy. After discharge, the dose of prednisolone tablets was modified to 20 mg (per os, once a day) and subsequently reduced to 1 tablet per week until discontinuation. The patient was later switched to oral antitumor therapy with anlotinib. Since then, no AIHA recurrence or tumor progression has been observed.

## 3. Discussion

### 3.1. Hematologic irAEs

Hematologic adverse effects are commonly induced by chemotherapy and primarily involve a reduction in blood cells, including white blood cells, neutrophils, platelets, and red blood cells, collectively known as myelosuppression. Although some drugs are associated with the development of AIHA,^[8]^ chemotherapy-induced AIHA is extremely uncommon. While cases of immune hemolytic anemia have been reported with fluorouracil analogs,^[9–11]^ such cases have not been observed with TGOPC.

Of all irAEs, hematologic irAEs are uncommon and often manifest as neutropenia, thrombocytopenia.^[12]^ Immunotherapy-related AIHA (ir-AIHA) should be diagnosed based on the evidence of hemolysis and a positive direct antiglobulin test. Most importantly, the condition should occur after the patient has been treated with an ICI and other causes have been ruled out, such as a delayed hemolytic transfusion reaction.^[13,14]^

A limitation of this case is the difficulty in truly identifying which drug is causing AIHA. It is also possible that the 2 drugs acted in concert to elicit this adverse effect. However, it is certain that AIHA occurred after the use of anti-PD-1 therapy in this patient. And based on the patient’s history of the successful administration of TGOPC after radiotherapy for ESC and the hematologic toxicity profile of anti-PD-1drugs, we considered that the patient’s AIHA was related to sintilimab, so the diagnosis of ir-AIHA was made.

### 3.2. Case reports of ir-AIHA

Existing case reports of ir-AIHA were collected from the literature, often reporting from various countries around the world, shown in Table 2 for details. The immunotherapy agents used primarily included pembrolizumab,^[15–19]^ nivolumab,^[20–26]^ ipilimumab,^[21–23]^ and atezolizumab,^[27–29]^ and the cancers involved were melanoma,^[15,16,20–23]^ lung adenocarcinoma,^[17,18,24,25,27]^ lymphoma,^[26]^ hepatocellular carcinoma,^[29]^ breast cancer,^[28]^ and upper urinary tract cancer.^[19]^ In these cases, ir-AIHA occurred at various stages of immunotherapy, including after the completion of treatment. Diagnosing ir-AIHA is generally straightforward when typical symptoms are present, such as anemia and jaundice, supported by appropriate laboratory tests. However, early detection and diagnosis are crucial. Additionally, the occurrence of ir-AIHA was often accompanied by other irAEs, such as pancytopenia,^[15]^ thrombocytopenia (Evan syndrome),^[18,19,22,29]^ primary hypothyroidism,^[23]^ cholangitis,^[16]^ and hemophagocytic lymphohistiocytosis.^[27]^ In terms of AIHA treatments, these cases received pharmacologic therapy with corticosteroids (e.g., prednisolone or methylprednisolone) and emergency treatment with red blood cell transfusions. In some cases, concomitant plasma exchange,^[17]^ rituximab,^[21,24]^ and cyclosporine A^[23]^ therapies were also administered. All cases of AIHA were resolved after treatment. However, 1 patient died due to comorbid atrial fibrillation and refusal of intubation.^[25]^ These findings underscore the need for the long-term monitoring of irAEs during ICI immunotherapy and demonstrate that effective and timely treatments are important to achieve favorable outcomes.

**Table 2:** Reported cases of ir-AIHA.

Author	Drugs for treatment	Cancer type	Patient age/gender	Immunotherapy	Combination drugs	Concurrent irAEs	Timing of AIHA	Salvage treatment	Outcome and follow-up
Ni et al 2019^[15]^	Pembrolizumab	Melanoma	67/Male	Pembrolizumab as first-line treatment	No	Pancytopenia	After 8th cycle from 1st dose	Methylprednisone 1 g dose and red cells transfusion	Improvement of AIHA, immunotherapy was discontinued
Williams et al 2019^[16]^	Pembrolizumab	Melanoma	81/Female	Pembrolizumab as first-line therapy (140 mg or 150 mg every 3 wk for 2 yr)	No	Cholangitis	6 wk after the end of immunotherapy	Intravenous methylprednisolone, oral prednisolone, red cells transfusion	Improvement of AIHA
Baek et al 2021^[17]^	Pembrolizumab	Lung adenocarcinoma	70/Male	Pembrolizumab as first-line therapy	Pemetrexed and cisplatin	No	2 wk after treatment	Prednisolone 2 mg/kg and plasma exchange 5 times every other day	After AIHA was cured, chemotherapy was continued and no hemolysis occurred
Zhang et al 2023^[18]^	Pembrolizumab	Lung adenocarcinoma	71/Male, with a history of AIHA	Pembrolizumab (a single dose of 200 mg) as first-line therapy	No	Thrombocytopenia (Evan syndrome), diarrhea, myocarditis, and acute kidney injury	10 d after treatment	Methylprednisolone 2 mg/kg for 3 d, followed by oral prednisone 60 mg	Hemolysis improved, palliative care without further treatment for cancer, and he eventually died within 3 mo
Kakita et al 2023^[19]^	Pembrolizumab	Upper urinary tract cancer	56/Male	Pembrolizumab as second-line therapy after chemotherapy	No	Thrombocytopenia (Evan syndrome)	After 26 mo (33 cycles) of treatment	Oral prednisolone 35 mg/d, red blood cells and platelet transfusions	Hemolysis improved
Carbó-Bagué et al 2021^[20]^	Nivolumab	Melanoma	62/Male	Nivolumab 3 mg/kg every 2 wk	No	No	After the 3rd cycle	Methylprednisolone 1 mg/kg, red blood cells transfusions	Hemolysis improved, subsequent use of ipilimumab after recurrence and no irAEs were found
Khan et al 2017^[21]^	Nivolumab and ipilimumab	Melanoma	43/Female	Immunotherapy as first-line therapy	No	No	After 2 cycles of treatment	Methylprednisolone 1000 mg daily for 3 d, Rituximab 375 mg/m^2^ weekly for 4 wk	AIHA recurred after the original immunotherapy
Fetter et al 2024^[22]^	Nivolumab and ipilimumab	Melanoma	64/Female	Nivolumab (1 mg/kg) and ipilimumab (3 mg/kg), every 3 wk	No	Thrombocytopenia	13 mo	Oral prednisolone 80 mg, red blood cell transfusion	Pembrolizumab was switched and AIHA occurred again
Olson et al 2020^[23]^	Nivolumab and ipilimumab	Melanoma	29/Female	Nivolumab (1 mg/kg) and ipilimumab (3 mg/kg), every 3 wk	No	Hypothyroidism	3 wk after 4th cycles	Intravenous corticosteroids，red blood cells, cyclosporine A 5 mg/kg/d	Hemolysis improved, immunotherapy was discontinued
Shaikh et al 2018^[24]^	Nivolumab	Lung adenocarcinoma	78/Male	Nivolumab 3 mg/kg, every 2 wk	No	No	After 39 cycles	Prednisone 1 mg/kg/d, conjunction with weekly rituximab for 4 doses	Hemolysis improved, Nivolumab was discontinued with stable lung cancer
Palla et al 2016^[25]^	Nivolumab	Lung adenocarcinoma	70/Male	Nivolumab 3 mg/kg, every 2 wk	No	Atrial fibrillation	3 d after the 2nd dose	Prednisone 1.5 mg/kg	The patient developed respiratory distress and expired
Tardy et al 2016^[26]^	Nivolumab	Hodgkin lymphoma	75/Female	Nivolumab (3 mg/kg，every 2 wk) as a third-line treatment	No	No	After 2 cycles of treatment	Prednisone 2 mg/kg, red blood cells	Hemolysis improved, continued to receive 6 cycles of nivolumab without recurrence of AIHA
Endo et al 2021^[27]^	Atezolizumab	Lung adenocarcinoma	65/Female	Atezolizumab as first-line therapy	Carboplatin and nab-paclitaxel	Hemophagocytic lymphohistiocytosis	After 1st cycle of treatment	Prednisolone 1 mg/kg/d	Hemolysis improved, chemotherapy and immunotherapy were not continued
Younce et al 2020^[28]^	Atezolizumab	Breast cancer	59/Female	Atezolizumab (840 mg, day 1 and day 14, every 4 wk)	Nab-paclitaxel (100 mg/m^2^)	No	Cycle 1, day 21	Prednisone taper 80 mg daily, folic acid, iron sucrose, and darbepoetin alfa	Hemolysis improved, the original regimen was continued without recurrence of AIHA
Fukushima et al 2023^[29]^	Atezolizumab	Hepatocellular carcinoma	86/Male	Atezolizumab as first-line therapy	Bevacizumab	Thrombocytopenia (Evan syndrome)	After 3 cycles of treatment	Prednisolone	Hemolysis improved, best supportive care

irAEs = immune-related adverse events, ir-AIHA = immunotherapy-related AIHA.

Most patients opted to discontinue immunotherapy after the resolution of ir-AIHA, and none of them experienced a recurrence of the condition. However, those who chose to continue immunotherapy had varying outcomes. Two female melanoma patients developed AIHA following treatment with nivolumab + ipilimumab. One patient continued with the original immunotherapy regimen,^[21]^ while the other switched to pembrolizumab.^[22]^ Both patients experienced a recurrence of AIHA. Additionally, 1 melanoma patient who switched to ipilimumab after experiencing an AIHA on nivolumab did not develop further irAEs.^[20]^ Similarly, a lymphoma patient who received nivolumab^[26]^ and a breast cancer patient^[28]^ who continued with the original atezolizumab regimen did not experience a recurrence of AIHA. As another limitation, we cannot currently explain the discrepancy in outcomes and still lack predictive factors for ir-AIHA occurrence and recurrence. Therefore, caution should be taken when continuing immunotherapy after ir-AIHA.

### 3.3. Ir-AIHA treatments

Corticosteroids are the first-line treatment for AIHA. Predniso(lo)ne is started at a dose of 1–2 mg/kg daily before adjusting depending on the patient’s conditions. Rituximab is a second-line treatment for AIHA. Third-line treatments include splenectomy or other immunosuppressants, such as azathioprine, cyclosporine, and mycophenolate.^[14,30]^ Prednisone(lo)ne in combination with rituximab is more effective in the treatment of AIHA.^[31]^ Although rituximab is not currently approved for use in AIHA, the early addition of rituximab should be considered in severe cases and in the absence of a rapid response to steroids. Blood transfusion is a mandatory measure in emergency situations, while immunoglobulin and plasma exchange can also be considered.^[14]^

Earlier studies have found that 39.7% of AIHA cases respond to intravenous immunoglobulin infusion^[32]^ at a dose of 0.4 g/kg/d for 5 days. Hence, it is recommended for use in critically ill or coinfected patients.^[30]^ In the latest consensus recommendations,^[33]^ corticosteroids and/or intravenous immunoglobulin are recommended for the first-line treatment of Evan syndrome with thrombocytopenia. Given that our AIHA patient was of advanced age, had a high number of comorbidities, and presented with severe conditions, we used methylprednisolone and immunoglobulin as first-line treatments and achieved satisfactory outcomes.

Kinase inhibitors, such as the Janus kinase and Bruton tyrosine kinase inhibitors, have shown promising therapeutic effects in other irAEs, such as immune-associated enteritis and myocarditis,^[34]^ and have demonstrated synergistic antitumor effects with ICIs. However, no large-cohort studies have been conducted to evaluate the use of this regimen in ir-AIHA, warranting further investigation.

### 3.4. Adverse effects of sintilimab

In the ORIENT-15 study,^[5]^ irAEs were significantly higher in the sintilimab plus chemotherapy group compared to the placebo plus chemotherapy group (47% vs 24%). Common AEs and their incidences in the sintilimab plus chemotherapy group were rash (13%), hypothyroidism (13%), and hyperthyroidism (6%). Furthermore, 10% of the patients developed grade ≥ 3 irAEs. Rare sintilimab-induced irAEs that have been reported include acute erosive hemorrhagic gastritis,^[35]^ cystitis/ureteritis,^[36]^ autoimmune diabetes,^[37]^ myocarditis,^[38–40]^ histiocytic necrotizing lymphadenitis,^[41]^ and inflammatory myopathy.^[42]^ This paper is the first to report on sintilimab-induced AIHA.

## 4. Conclusions

Although ir-AIHA is uncommon, it is often severe and may even be life-threatening. Therefore, we should remain vigilant throughout the course of tumor immunotherapy. In this context, the detection and recognition of early symptoms, efficient differential diagnosis, multidisciplinary consultations, and timely and effective treatments are vital for patient outcomes.

## Acknowledgments

We thank our colleagues in the Department of Hematology for their consultation assistance.

## Author contributions

**Conceptualization:** Zijian Qiu, Mixue Sun, Xiaoping Zhu.

**Funding acquisition:** Xiaoping Zhu.

**Investigation:** Zijian Qiu, Mixue Sun, Chunyan Dai.

**Project administration:** Xiaoping Zhu.

**Resources:** Chunyan Dai.

**Writing – original draft:** Zijian Qiu, Mixue Sun, Chunyan Dai.

**Writing – review & editing:** Zijian Qiu, Mixue Sun, Xiaoping Zhu.

## References

[1] Morgan E Soerjomataram I Rumgay H. The global landscape of esophageal squamous cell carcinoma and esophageal adenocarcinoma incidence and mortality in 2020 and projections to 2040: new estimates from GLOBOCAN 2020. Gastroenterology. 2022;163:649–58.e2. 35671803 10.1053/j.gastro.2022.05.054

[2] Shoji Y Koyanagi K Kanamori K. Immunotherapy for esophageal cancer: where are we now and where can we go. World J Gastroenterol. 2024;30:2496–501. 38817664 10.3748/wjg.v30.i19.2496 PMC11135418

[3] Sun JM Shen L Shah MA. Pembrolizumab plus chemotherapy versus chemotherapy alone for first-line treatment of advanced oesophageal cancer (KEYNOTE-590): a randomised, placebo-controlled, phase 3 study. Lancet. 2021;398:759–71. 34454674 10.1016/S0140-6736(21)01234-4

[4] Doki Y Ajani JA Kato K. Nivolumab combination therapy in advanced esophageal squamous-cell carcinoma. N Engl J Med. 2022;386:449–62. 35108470 10.1056/NEJMoa2111380

[5] Lu Z Wang J Shu Y. Sintilimab versus placebo in combination with chemotherapy as first line treatment for locally advanced or metastatic oesophageal squamous cell carcinoma (ORIENT-15): multicentre, randomised, double blind, phase 3 trial. BMJ. 2022;377:e068714. 35440464 10.1136/bmj-2021-068714 PMC9016493

[6] Ramos-Casals M Brahmer JR Callahan MK. Immune-related adverse events of checkpoint inhibitors. Nat Rev Dis Primers. 2020;6:38. 32382051 10.1038/s41572-020-0160-6 PMC9728094

[7] Delanoy N Michot JM Comont T. Haematological immune-related adverse events induced by anti-PD-1 or anti-PD-L1 immunotherapy: a descriptive observational study. Lancet Haematol. 2019;6:e48–57. 30528137 10.1016/S2352-3026(18)30175-3

[8] Maquet J Lafaurie M Michel M Lapeyre-Mestre M Moulis G. Drug-induced immune hemolytic anemia: detection of new signals and risk assessment in a nationwide cohort study. Blood Adv. 2024;8:817–26. 37782770 10.1182/bloodadvances.2023009801 PMC10874903

[9] Sideris S Loizidou A Georgala A. Autoimmune haemolytic anaemia in a patient treated with capecitabine. Acta Clin Belg. 2013;68:135–7. 23967725 10.2143/ACB.3149

[10] Zurita Saavedra AJ Navarro Garcia M Español I Fernández Ortega A. UFT-induced haemolytic anaemia. Cancer Chemother Pharmacol. 2001;47:280–1. 11320674 10.1007/s002800000195

[11] Yürek S Riess H Kreher S Dörken B Salama A. Fatal immune haemolysis due to antibodies to individual metabolites of 5-fluorouracil. Transfus Med. 2010;20:265–8. 20456688 10.1111/j.1365-3148.2010.01009.x

[12] Kramer R Zaremba A Moreira A. Hematological immune related adverse events after treatment with immune checkpoint inhibitors. Eur J Cancer. 2021;147:170–81. 33706206 10.1016/j.ejca.2021.01.013

[13] Hwang SR Saliba AN Wolanskyj-Spinner AP. Immunotherapy-associated autoimmune hemolytic anemia. Hematol Oncol Clin North Am. 2022;36:365–80. 35339260 10.1016/j.hoc.2021.11.002

[14] Jäger U Barcellini W Broome CM. Diagnosis and treatment of autoimmune hemolytic anemia in adults: recommendations from the first international consensus meeting. Blood Rev. 2020;41:100648. 31839434 10.1016/j.blre.2019.100648

[15] Ni D AlZahrani F Smylie M. AIHA and pancytopenia as complications of pembrolizumab therapy for metastatic melanoma: a case report. Case Rep Oncol. 2019;12:456–65. 31275137 10.1159/000500856 PMC6600028

[16] Williams H Aitchison R. Pembrolizumab-induced autoimmune haemolytic anaemia and cholangitis. BMJ Case Rep. 2019;12:e232505. 10.1136/bcr-2019-232505 PMC6904205 31811095

[17] Baek DW Chae YS. Pembrolizumab-related autoimmune hemolytic anemia in a patient with metastatic lung adenocarcinoma: a case report. Yeungnam Univ J Med. 2021;38:366–70. 33752275 10.12701/yujm.2021.00899 PMC8688779

[18] Zhang X Gao BX Guo CY Su T. A 71-year-old male with a life-threatening recurrence of hemolytic anemia, thrombocytopenia, and acute kidney injury after pembrolizumab therapy: a case report. BMC Geriatr. 2023;23:478. 37553570 10.1186/s12877-023-04181-w PMC10410872

[19] Kakita S Matsuo T Ohki M. Evans syndrome during pembrolizumab therapy for upper urinary tract cancer. IJU Case Rep. 2023;6:298–301. 37667757 10.1002/iju5.12609 PMC10475338

[20] Carbó-Bagué A Fort-Culillas R Pla-Juher H Rubió-Casadevall J. Nivolumab-induced autoimmune haemolytic anaemia and safety of subsequent use of ipilimumab: a case report. Case Rep Oncol. 2021;14:1289–94. 34720930 10.1159/000518530 PMC8460943

[21] Khan U Ali F Khurram MS Zaka A Hadid T. Immunotherapy-associated autoimmune hemolytic anemia. J ImmunoTher Cancer. 2017;5:15. 28239468 10.1186/s40425-017-0214-9 PMC5319184

[22] Fetter T Fietz S Bertlich M. Severe autoimmune hemolytic anemia following immunotherapy with checkpoint inhibitors in two patients with metastatic melanoma: a case report. Front Immunol. 2024;15:1342845. 38571955 10.3389/fimmu.2024.1342845 PMC10987708

[23] Olson DJ Rajagopal P Tjota MY Venkataraman G Luke JJ Gajewski TF. A case of dual-mechanism immune-related anaemia in a patient with metastatic melanoma treated with nivolumab and ipilimumab. J ImmunoTher Cancer. 2020;8:e000380. 32169870 10.1136/jitc-2019-000380 PMC7069276

[24] Shaikh H Daboul N Albrethsen M Fazal S. A case of autoimmune haemolytic anaemia after 39 cycles of nivolumab. BMJ Case Rep. 2018;2018:bcr2018224608. 10.1136/bcr-2018-224608 PMC5911145 29669775

[25] Palla AR Kennedy D Mosharraf H Doll D. Autoimmune hemolytic anemia as a complication of nivolumab therapy. Case Rep Oncol. 2016;9:691–7. 27920704 10.1159/000452296 PMC5126613

[26] Tardy MP Gastaud L Boscagli A Peyrade F Gallamini A Thyss A. Autoimmune hemolytic anemia after nivolumab treatment in Hodgkin lymphoma responsive to immunosuppressive treatment. A case report. Hematol Oncol. 2017;35:875–7. 27539158 10.1002/hon.2338

[27] Endo Y Inoue Y Karayama M. Marked, lasting disease regression and concomitantly induced autoimmune hemolytic anemia and hemophagocytic lymphohistiocytosis in a patient with lung adenocarcinoma and autoantibodies receiving atezolizumab plus chemotherapy: a case report. JTO Clin Res Rep. 2022;3:100263. 35005655 10.1016/j.jtocrr.2021.100263 PMC8718484

[28] Younce CM Lawton JM Patel DR. Atezolizumab-induced hemolytic anemia—a case report. J Oncol Pharm Pract. 2021;27:1026–8. 32951522 10.1177/1078155220957720

[29] Fukushima M Tajima K Sasaki R. Evans’ syndrome induced by atezolizumab plus bevacizumab combination therapy in advanced hepatocellular carcinoma. Clin J Gastroenterol. 2023;16:402–6. 36746879 10.1007/s12328-023-01767-0

[30] Barcellini W Fattizzo B. How I treat warm autoimmune hemolytic anemia. Blood. 2021;137:1283–94. 33512406 10.1182/blood.2019003808

[31] Michel M Terriou L Roudot-Thoraval F. A randomized and double-blind controlled trial evaluating the safety and efficacy of rituximab for warm auto-immune hemolytic anemia in adults (the RAIHA study). Am J Hematol. 2017;92:23–7. 27696475 10.1002/ajh.24570

[32] Flores G Cunningham-Rundles C Newland AC Bussel JB. Efficacy of intravenous immunoglobulin in the treatment of autoimmune hemolytic anemia: results in 73 patients. Am J Hematol. 1993;44:237–42. 8237993 10.1002/ajh.2830440404

[33] Fattizzo B Marchetti M Michel M. Diagnosis and management of Evans syndrome in adults: first consensus recommendations. Lancet Haematol. 2024;11:e617–28. 38968944 10.1016/S2352-3026(24)00144-3

[34] Henderson Berg MH Del Rincón SV Miller WH. Potential therapies for immune-related adverse events associated with immune checkpoint inhibition: from monoclonal antibodies to kinase inhibition. J ImmunoTher Cancer. 2022;10:e003551. 35086945 10.1136/jitc-2021-003551 PMC8796266

[35] Ai Q Chen W Li Y Li G. Upper gastrointestinal tract IrAEs: a case report about sintilimab-induced acute erosive hemorrhagic gastritis. Front Immunol. 2022;13:840916. 35720298 10.3389/fimmu.2022.840916 PMC9204206

[36] Tu L Ye Y Tang X. Case report: a case of sintilimab-induced cystitis/ureteritis and review of sintilimab-related adverse events. Front Oncol. 2021;11:757069. 35004277 10.3389/fonc.2021.757069 PMC8733470

[37] Yang J Wang Y Tong XM. Sintilimab-induced autoimmune diabetes: a case report and review of the literature. World J Clin Cases. 2022;10:1263–77. 35211559 10.12998/wjcc.v10.i4.1263 PMC8855200

[38] Liu X Zeng Z Cao J. Sintilimab-induced myocarditis in a patient with gastric cancer: a case report and literature review. J Cardiovasc Dev Dis. 2023;10:422. 37887869 10.3390/jcdd10100422 PMC10607029

[39] Ji H Wen Z Liu B Chen H Lin Q Chen Z. Sintilimab induced ICIAM in the treatment of advanced HCC: a case report and analysis of research progress. Front Immunol. 2022;13:995121. 36091070 10.3389/fimmu.2022.995121 PMC9458972

[40] Yin B Xiao J Wang X. Myocarditis and myositis/myasthenia gravis overlap syndrome induced by immune checkpoint inhibitor followed by esophageal hiatal hernia: a case report and review of the literature. Front Med (Lausanne). 2022;9:950801. 36457566 10.3389/fmed.2022.950801 PMC9705572

[41] Ren C Wang Y Yang X Tuo Y Li Y Gong J. Kikuchi disease: a case report about Sintilimab-induced Kikuchi histiocytic necrotizing lymphadenitis and literature review. Heliyon. 2024;10:e30608. 38742085 10.1016/j.heliyon.2024.e30608 PMC11089371

[42] Hong G Zhao H Yin Y. Sintilimab-induced inflammatory myopathy in a patient with esophageal cancer: a case report. Front Immunol. 2023;14:1253463. 37920461 10.3389/fimmu.2023.1253463 PMC10619899

